# Effects of Long-Term 50Hz Power-Line Frequency Electromagnetic Field on Cell Behavior in Balb/c 3T3 Cells

**DOI:** 10.1371/journal.pone.0117672

**Published:** 2015-02-19

**Authors:** Guang-Zhou An, Hui Xu, Yan Zhou, Le Du, Xia Miao, Da-Peng Jiang, Kang-Chu Li, Guo-Zhen Guo, Chen Zhang, Gui-Rong Ding

**Affiliations:** 1 Department of Radiation Medicine, Faculty of Preventive Medicine, The Fourth Military Medical University, Xi’an, Shaanxi province, China; 2 Radiological College of Taishan Medical College, Taishan, Shandong province, China; 3 China Academy of Telecommunication Research of Ministry of Industry and Information Technology, Beijing, China; Instituto Butantan, BRAZIL

## Abstract

Power-line frequency electromagnetic field (PF-EMF) was reported as a human carcinogen by some epidemiological research, but the conclusion is lack of robust experiment evidence. To identify the effects of long-term PF-EMF exposure on cell behavior, Balb/c 3T3 cells in exponential growth phase were exposed or sham-exposed to 50 Hertz (Hz) PF-EMF at 2.3 mT for 2 hours (h) one day, 5 days every week. After 11 weeks exposure, cells were collected instantly. Cell morphology was observed under invert microscope and Giemsa staining, cell viability was detected by 3-(4, 5-dimethylthiazol-2-yl)-2, 5-diphenyltetrazolium bromide (MTT) assay, cell cycle and apoptosis was examined by flow cytometry, the protein level of Proliferating Cell Nuclear Antigen (PCNA) and CyclinD1 was detected by western blot, cell transformation was examined by soft agar clone assay and plate clone forming test, and cell migration ability was observed by scratch adhesion test. It was found that after PF-EMF exposure, cell morphology, apoptosis, cell migration ability and cell transformation didn’t change. However, compared with sham group, cell viability obviously decreased and cell cycle distribution also changed after 11 weeks PF-EMF exposure. Meanwhile, the protein level of PCNA and CyclinD1 significantly decreased after PF-EMF exposure. These data suggested that although long-term 50Hz PF-EMF exposure under this experimental condition had no effects on apoptosis, cell migration ability and cell transformation, it could affect cell proliferation and cell cycle by down-regulation the expression of PCNA and CyclinD1 protein.

## Introduction

Nowadays, human living environment was surrounded by power-line frequency electromagnetic field (PF-EMF) mainly generated by electrical equipment and power lines. As the intensity of PF-EMF becomes more and more high, and the exposure time of human beings increases, the potential harmful effects of PF-EMF on human health, especially its carcinogenic risk, have been paid much attention. To evaluate the potential carcinogenic effects of PF-EMF, lots of epidemiological researches have been carried out and focused primarily on leukemia development due to residential exposure on children [1] and adults [2], occupational exposure in adults [3], brain tumors [4] and breast tumors [5]. However, no conclusions have been drawn due to some negative reports [6, 7] and methodological deficiencies in epidemiological research. Apart from epidemiological research, long-term bioassays have been performed in which the potential oncogenicity in experimental animals exposed to ELF electromagnetic fields including PF-EMF was evaluated, and no consistent results were found [8,9,10]. Due to the principle of prudence, although there is limited evidence for the carcinogenicity of ELF magnetic fields, International Agency for Research on Cancer classified it as “potential human carcinogens (2B)”in 2002.

At present, there is still not adequate and robust experimental evidence to support the viewpoint that PF-EMF is a carcinogen. Consistent and independently replicated laboratory evidence to support a causative relationship between exposure to PF-EMF and the tumor risk has not been obtained.

It is well known that cancer stems from the transformation of normal cells, after transformation, the cell biological property would change and obtain characteristics of cancer cell [11]. It’s reported that ELF could influence cell biological property such as proliferation [12] and apoptosis [13] in some short-term PF-EMF exposure conditions, however, the effects of PF-EMF exposure on biological property of Balb/c 3T3 cells which are often used to test environmental potential carcinogen have not been reported. To explore the effects of long-term PF-EMF exposure on cell biological property and whether PF-EMF could result in cell transformation, Balb/c 3T3 cells were used in this study, and cell behavior including cell morphology, cell viability, cell cycle, apoptosis, cell migration and transformation ability were observed after 11 weeks PF-EMF exposure.

## Materials and Methods

### Cell culture

The Balb/c mouse embryo fibroblasts (Balb/c 3T3 cells), rat glioma cells (C6 cells) and human esophageal squamous carcinoma cells (KYSE150 cells) were purchased from shanghai cell bank of Chinese academy of sciences. Balb/c 3T3 and C6 were adherent growing, while KYSE150 cells were anchorage-independent growing. They were all maintained in Dulbecco’s modified Eagle’s medium (DMEM; Invitrogen corporation, Carlsbad, CA, USA), with 10% fetal bovine serum (FBS; Gibco, Grand Island, NY, USA) and were cultured in a humidified atmosphere containing 5% CO_2_ incubator at 37°C.Cells were digested by 0.25% trypsin solution (Invitrogen) in the exponential growth stage and were then passaged into T25 flasks.

### PF-EMF Exposure Setup

**Fig 1:**
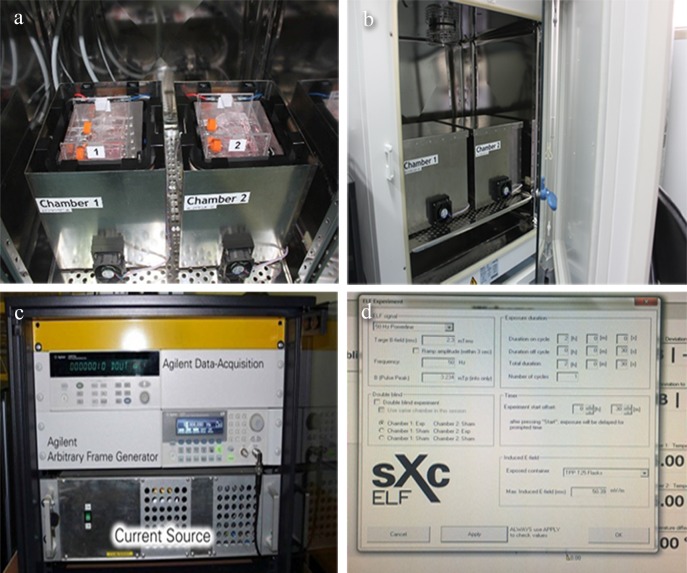
The PF-EMF exposure system. (a) mu-metal box; (b) incubator; (c) current source; (d) arbitrary function generator controlled by a computer.

The PF-EMF setup (sXc-ELF, IT’IS foundation, Zurich, Switzerland) consists of two four-coil systems, each of which are placed inside a mu-metal box labelled “chamber 1” or “chamber 2” (Fig. 1A). Mu-metal is a special alloy for high magnetic shielding. The current in the bifilar coils can be switched parallel for field exposure or non-parallel for sham control. Both systems fit inside a commercial incubator (Fig. 1B) to ensure the constant environmental condition that a temperature is 37°C and atmosphere consists of 95% air/5% CO_2_ and 100% relative humidity. In addition, the temperature is monitored at the location of the flasks during exposure with Pt100 probes. Two fans per box are mounted to guarantee enough atmospheric exchange of the exposure chambers.

The setup has been optimized for homogeneous field distribution, maximum field strength, minimum temperature increase and minimum vibrations. A current source (Fig. 1C) based on four audio amplifiers was developed which allows magnetic fields up to 3.5 mT. The field can be arbitrary varied in the frequency range from 3–1250 Hz by a computer controlled arbitrary function generator (Fig. 1D). The fields as well as all sensors are continuously monitored.

The generated magnetic field used in this work was power-line signal with frequency of 50 Hz and B-field root mean square (rms) strength 2.3 mT. The non-uniformity of the magnetic field was less than 4%. The computer controls and monitors the entire setup.

### PF-EMF exposure

Balb/c 3T3 cells in exponentially growing phase were used for exposure or sham exposure. The cell density was 1×10^4^ cells in each flask. Cells were continuously exposed to 50 Hz PF-EMF at 2.3 mT for 2 h one day, 5 days every week (from Monday to Friday), totally 11 weeks. Medium was changed once a week. Cells were passaged after five-day exposure every week, and the cell density was kept at 1×10^4^ cells. After 11 weeks exposure to PF-EMF, cells were harvested instantly.

### Cell morphology observation

Cell morphology was observed under invert microscope every week. In addition, Giemsa staining was carried out after 11 weeks exposure. Photographs were taken when cells reached 80%~90% confluence in sham group and exposure group. Cells were rinsed by Phosphate-buffer saline (PBS) twice after medium were removed in T25 flask, then fixed with 5 ml 75% methanol for 15 minutes (min) and stained with Giemsa (Baso Diagnostics Inc, Zhuhai, Guangdong Province, China) for 20 min at Room Temperature (RT, 25°C). Afterward, cells were washed and the cell morphology were observed under inverted microscope (Nikon Corporation, Tokyo, Japan).

### MTT assay

Cells were seeded into a flat-bottom, 96-well plate (1200cells/well) in quintuplicate. On the third day after seeding, 20 μl MTT (5 mg/ml; Sigma-Aldrich, Saint Louis, Missouri, USA) was added to each well and further incubated for 4 h. Cells were then solubilized in 150 μl Dimethyl sulfoxide (Sigma). The absorbance reading was obtained using 96-well spectrophotometer (Bio-Rad, Hercules, CA, USA). The viability histogram were created by plotting the average of quintuplicate values calculated by optical density measurements at 490 nm.

### Cell cycle analysis by flow cytometry

After exposure, cells were harvested, and then washed twice using PBS, and fixed in 70% ethanol at 4°C overnight. Then cells were resuspended in PBS containing 0.01% RNase (Sigma) and incubated with 0.5% propidium iodide (BD, Franklin Lakes, NJ, USA) at RT for 1 h and were analyzed by flow cytometry using a FACSCalibur Flow Cytometer at 488 nm (BD). The percentage of cells in different phases of the cell cycle was calculated by MultiCycle (DeNovo software, Thornhill, ON, Canada).

### Apoptosis assay by flow cytometry

The assay followed instructions of Fluorescein Isothiocyanate Annexin V Apoptosis Detection Kit Ⅱ(BD). Cells were washed twice with ice-cold PBS and then resuspended in 1× Binding Buffer at a concentration of 1×10^6^ cells/ml. 100 μl of the solution (1×10^5^ cells) was transferred to a 5 ml culture tube. 5 μl of Fluorescein Isothiocyanate Annexin V and 5 μl propidium iodide was added to the tube. Cells were gently vortexed and incubated for 15 min at RT in the dark, then 400 μl of 1× Binding Buffer was added to each tube without washing and analyzed using FACSCalibur Flow Cytometer (BD) as soon as possible (within 1 h). Balb/c 3T3 cells treated with 12 Gy X-ray radiation were used as positive control.

### Western Blot

Cell extracts were prepared by lysing the ice-cold PBS washed cells in the designated times by using 100 μL lysis buffer (50 mM hydroxyethyl piperazine ethanesulfonic acid, pH 7.4; 5 mM 3-[(3-cholamidopropyl)dimethyl-ammonio]-1-propanesulfonate; 5 mM dithiothreitol) at 4°C for 15 min. Extracts were then centrifuged at 14,000 g in a microfuge at 4°C, and supernatants were transferred to fresh tubes. Protein concentration were quantified by Bradford assay method using the Bio-Rad Dc System (Bio-Rad). Equal amounts of protein (20 μg) were dissolved in lysis buffer, the samples were boiled for 5 min. Proteins were separated on standard SDS-polyacrylamide gel (SDS-PAGE), and transferred onto Polyvinylidene Difluoride membranes (Millipore, Billerica, MA, USA). After blocking with 5% skim milk in Tris-buffered saline/Tween, the membrane was incubated with PCNA (1:500 dilution, Abcam, Cambridge, MA, USA)、CyclinD1 (1:500 dilution, Abcam) and β-actin antibody (1:500 dilution, Abcam) for 18 h at 4°C, then washed and incubated with anti-rabbit secondary antibody conjugated with Horseradish Peroxidase (1:8000 dilution, Abcam) for 1 h at RT. Enhanced Chemiluminescence (Bio-Rad) was used and the signal was visualized by adding luminal substrate to the blots and exposing to film.

### Scratch adhesion test

Cells were plated in 24-well plates and grew to confluence to form a monolayer. Wounds were created by scraping the cell monolayer with a 200 μL micropipette tip. After rinsing with PBS three times, the wells were refilled by DMEM with 1.5% FBS. The wounds were photographed at the beginning and 12 h later, and the area of wounds was measured by National Instrument Vision Assistant 8.5 software. Healing area was calculated by the formula: wound healing area = 0 h wound area—12 h wound area.

### Soft agar clone assay

The malignant transformation system of in vitro Balb/c 3T3 cells is often used in cell transformation experiment [14, 15], and it is usually adopted as a classic method in determining carcinogens or cancer promoter. Firstly, 1.2% and 0.6% agarose (Sigma) were prepared with sterile H_2_O and stored in a 4°C refrigerator. Secondly, 2×DMEM culture medium containing 20% FBS was prepared and kept within 37°C water bath. Then, 0.7 ml 1.2% agarose and 0.7 ml 2×DMEM were mixed uniformly in a tube and poured into a 6-well plate to be used as base agar. It was allowed to solidify at RT. Next, Balb/c 3T3 and KYSE150 cells (used as a positive control) were digested, centrifuged and resuspended in DMEM medium to form single cell suspension. The concentrations were adjusted to 1×10^4^ cells/ml. After that, 0.5 ml 2×DMEM culture medium and 0.5 ml 0.6% agarose were mixed uniformly, and further inoculated with 1×10^3^ cells/0.1 ml of each group to form top agar and subsequently added to the base agar to form two-layer agar. It was allowed to solidify at RT. Finally, the 6-well plates were placed in a 37°C, 5% CO_2_ incubator and observed with an inverted microscope (Olympus, Tokyo, Japan) every day.

### Plate clone assay

Balb/c 3T3 and C6 cells (used as a positive control) were plated in triplicate at 100 cells per well in 6-well plates and cultured in DMEM medium supplemented with 10% FBS. When visible colonies formed on the seventh day after plating they were washed twice by PBS and fixed in 75% methanol for 15 min, and stained with Giemsa for 20 min at RT. Afterward, the dye was washed off and colonies were observed by inverted microscope.

### Statistical analysis

All experiments were conducted at least in triplicate, and data analysis was performed using SPSS software. Statistical significance was assessed using the Student t-test. P < 0.05 was defined to be a statistically significant difference between two groups.

## Results

### Effects of PF-EMF exposure on cell morphology

**Fig 2:**
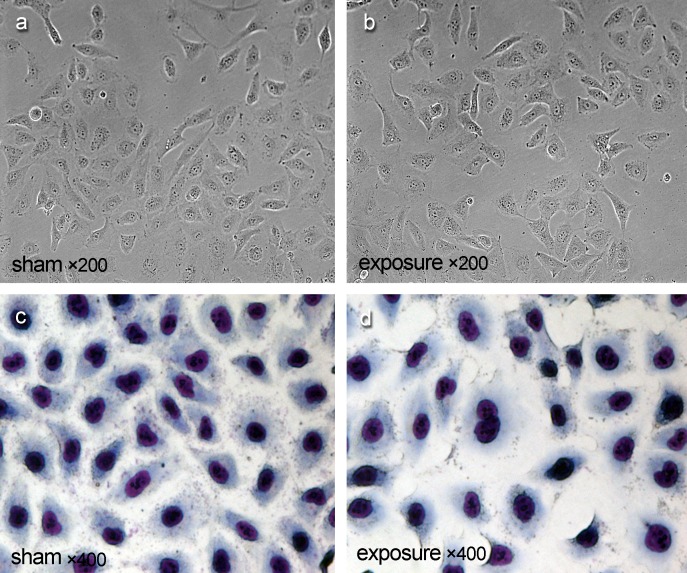
The cell morphology and nuclear morphology of Balb/c cells after 11 weeks PF-EMF exposure.

Compared with sham group (Fig. 2A, 2C), Balb/c 3T3 cells didn’t show any change in cell morphology and nuclear morphology under invert microscope (Fig. 2B) and by Giema staining (Fig. 2D) after 11 weeks exposure to PF-EMF.

### Effects of PF-EMF exposure on cell viability

**Fig 3:**
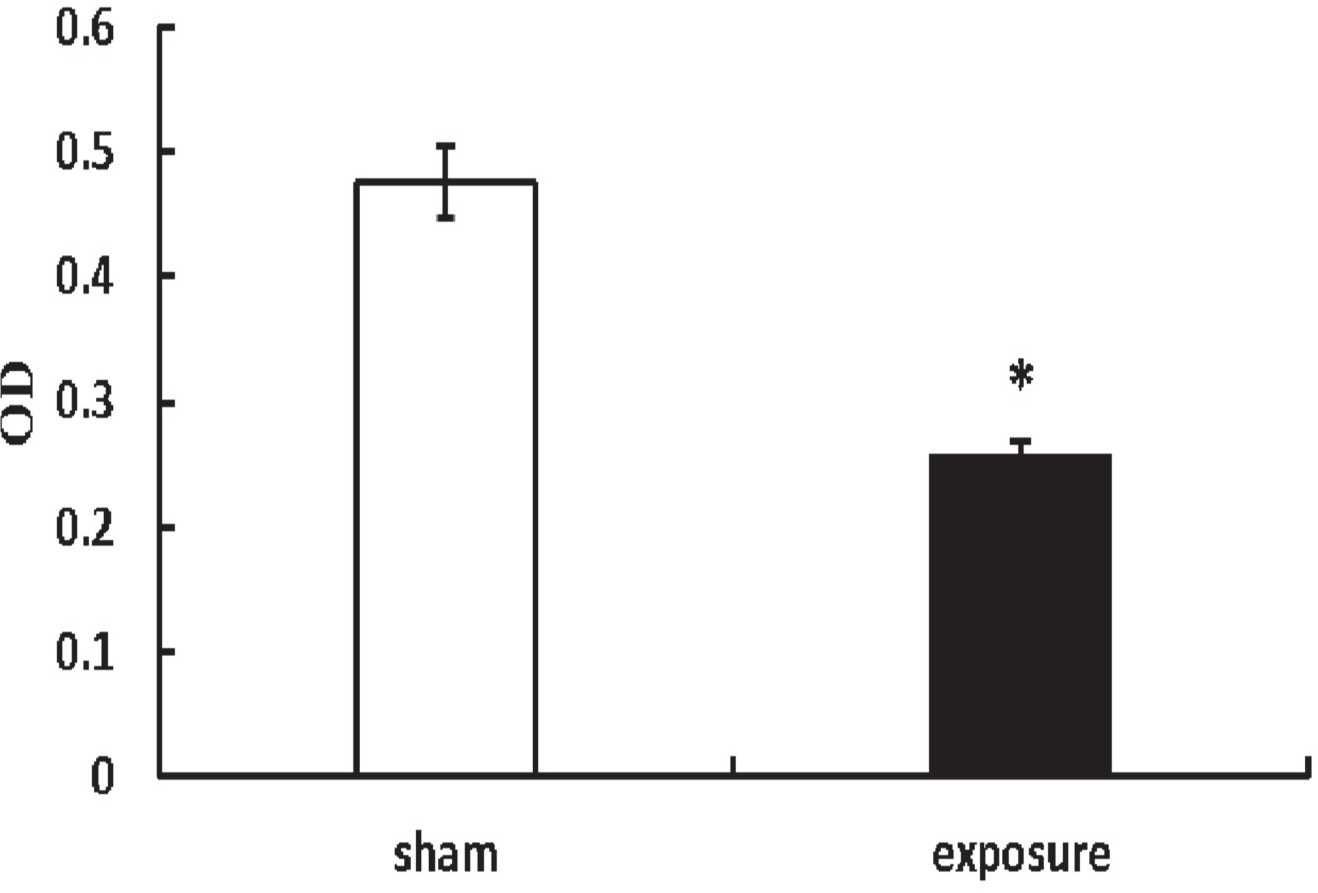
The cell viability of Balb/c 3T3 cells after 11 weeks PF-EMF exposure. *vs. sham group, P<0.05.

MTT assay results showed that the viability of Balb/c cells significantly decreased (OD value: 0.26±0.01) after 11 weeks exposure to PF-EMF (p<0.05) compared with sham group (OD value: 0.48±0.03) (Fig. 3).

### Effects of PF-EMF exposure on cell cycle

**Fig 4:**
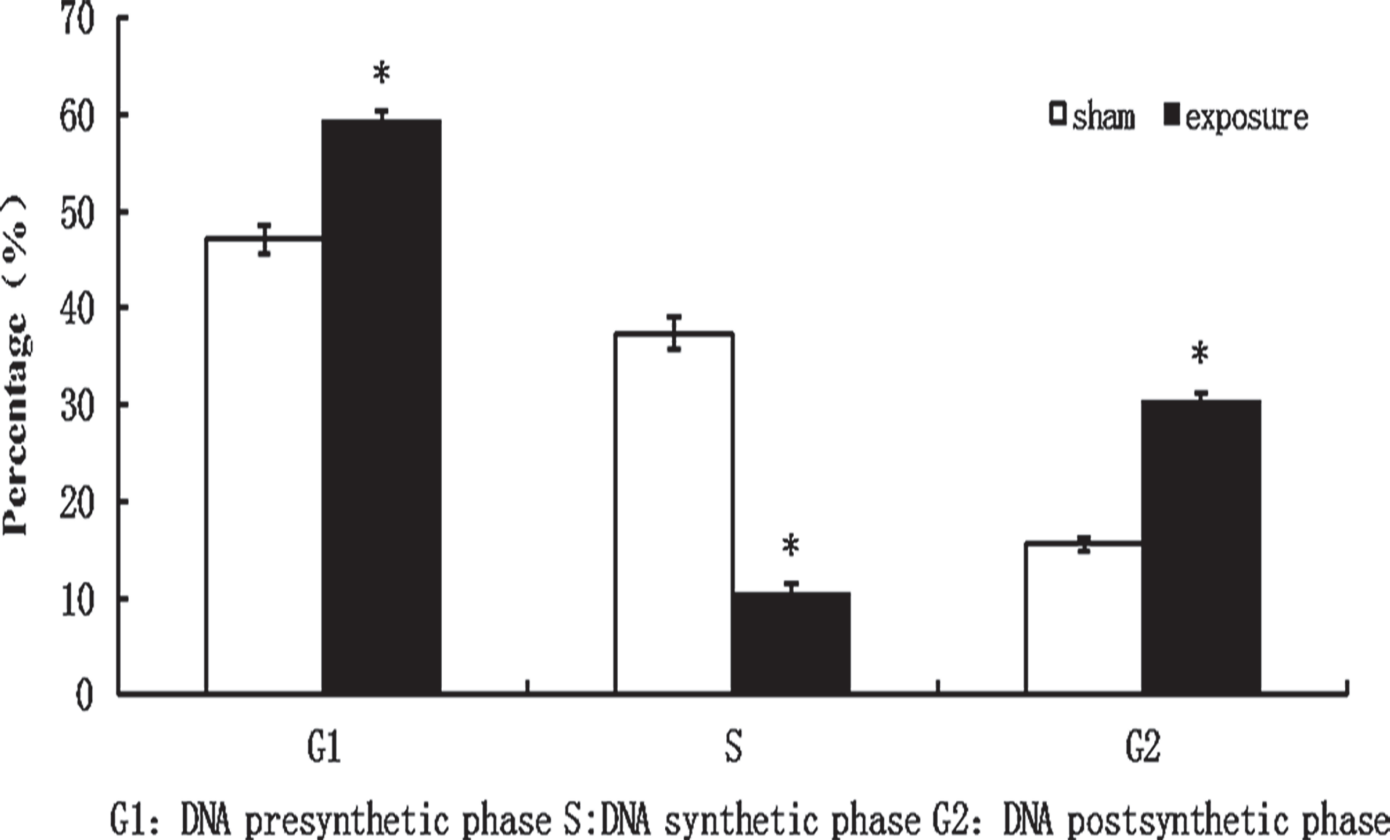
The cell cycle distribution of Balb/c 3T3 cells after 11 weeks PF-EMF exposure. *vs. sham group, P<0.05.

Compared with sham group (cells in S phase: 37.34%±1.65%; cells in G_2_ phase: 15.57%±0.71%), the percentage of 11 weeks PF-EMF exposed cells in S phase significantly decreased (10.38%±1.13%) and cells in G_2_ phase significantly increased (30.25%±0.94%) (p<0.05) (Fig. 4).

### Effects of PF-EMF exposure on protein expression

**Fig 5:**
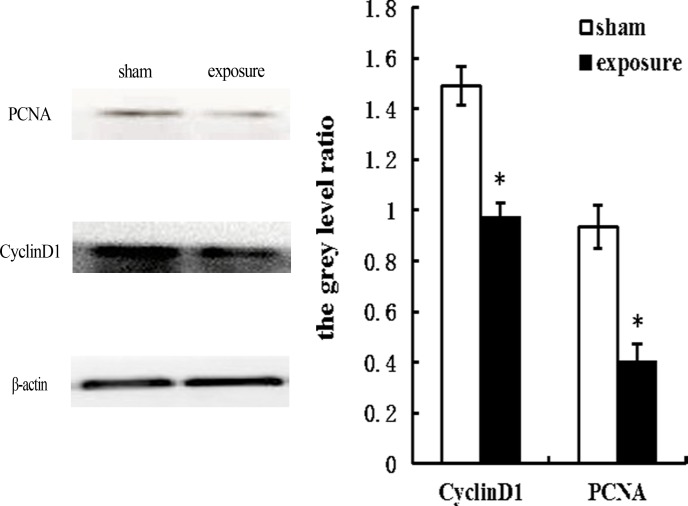
The protein level of PCNA and CyclinD1 in Balb/c 3T3 cells after 11 weeks PF-EMF exposure. β-actin was used as the internal control. The grey level ratio = the grey level of targeted protein/the grey level of β-actin. *vs. sham group, P<0.05.

Compared with sham group, the protein levels of PCNA and CyclinD1 significantly decreased after 11 weeks exposure to PF-EMF (p<0.05) (Fig. 5).

**Fig 6:**
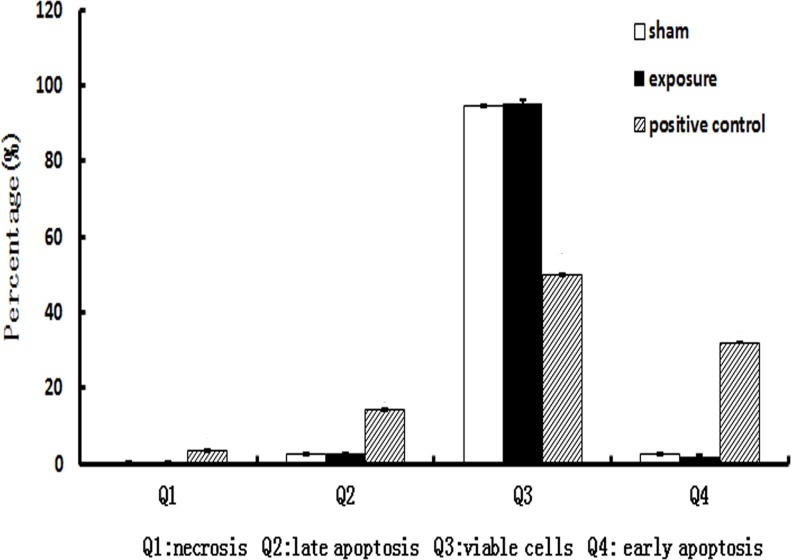
The apoptosis level of Balb/c 3T3 cells after 11 weeks PF-EMF exposure.

**Effects of PF-EMF exposure on apoptosis**. In case of apoptosis, it was found that compared with sham group (Q4: 2.6%±0.1%; Q2: 2.6%±0.2%), both the early apoptosis level (Q4: 1.8%±0.4%) and late apoptosis level (Q2: 2.6%±0.3%) didn’t change in Balb/c 3T3 cells after 11 weeks exposure to PF-EMF (p>0.05). High-level early apoptosis and late apoptosis of Balb/c 3T3 cells were found in positive control group (Fig. 6).

### Effects of PF-EMF exposure on migration ability

**Fig 7:**
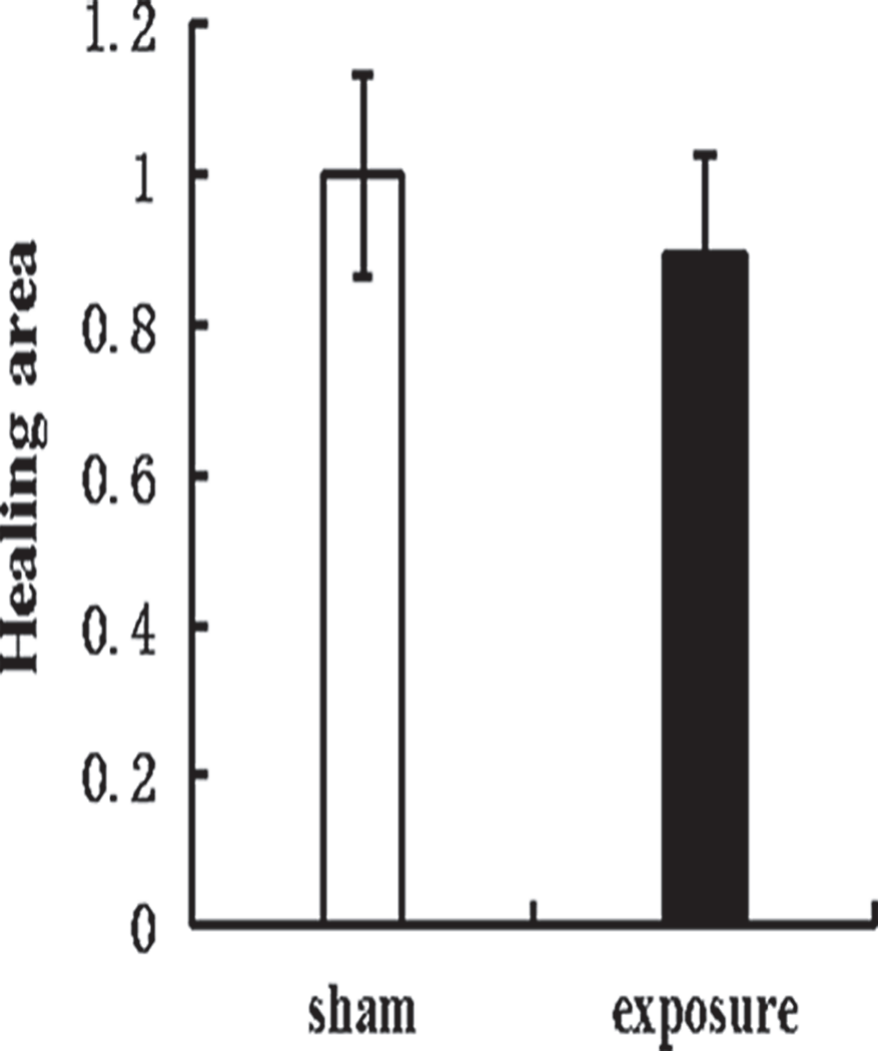
The cell migration rate of Balb/c 3T3 cells after 11 weeks PF-EMF exposure. Wound healing area in exposure group was denoted by the healing area ratio of two groups.

In scratch adhesion test, 11 weeks exposure to PF-EMF did not change cell migration rate of Balb/c 3T3 cells (p>0.05), compared with sham group (Fig. 7).

### Effects of PF-EMF exposure on clone forming in soft agar

**Fig 8:**
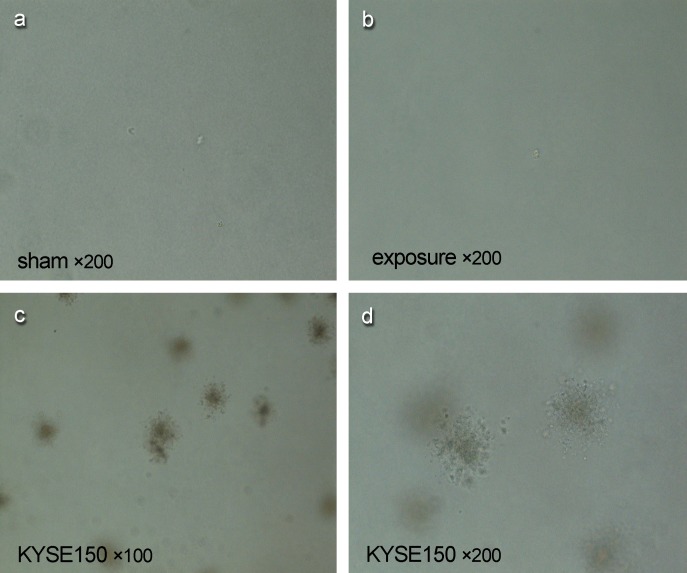
The soft agar clone formation of Balb/c 3T3 cells after 11 weeks PF-EMF exposure.

In soft agar clone assay, no clone of Balb/c 3T3 cells was found after 11 weeks exposure to PF-EMF (Fig. 8B), while in positive control group, lots of clones were found in KYSE150 cells under the same experimental condition (Fig. 8C,D).

### Effects of PF-EMF exposure on plate clone forming

**Fig 9:**
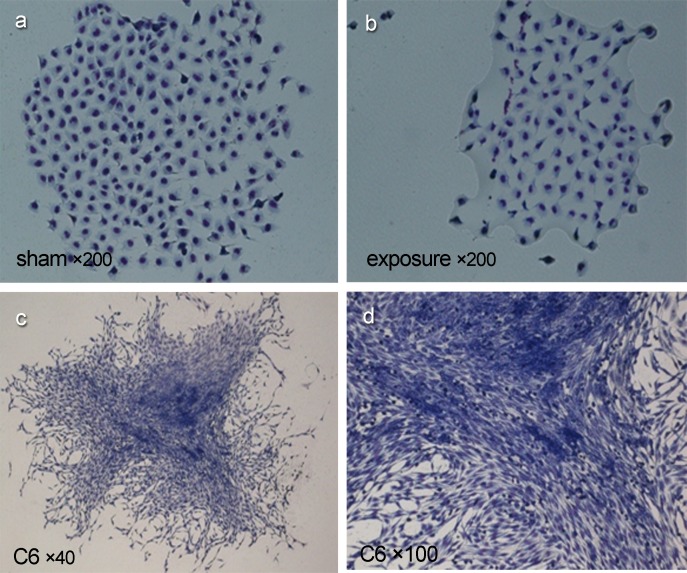
The plate clone formation of Balb/c 3T3 cells after 11 weeks PF-EMF exposure.

In plate clone forming test, no malignant transformation focus was found in Balb/c 3T3 cells after 11 weeks exposure to PF-EMF (Fig. 9B), while typically malignant clone characteristics including alkaliphilic deep dying, compact multiple cell layers, free orientation, invasive growth and spindle morphology was seen in clones of C6 cells in positive control group (Fig. 9C,D).

## Discussion

It was reported that ELF could influence the cell morphology through altering cytoskeletal organization [16], and the cell morphology has a close relation with cell biological property. For example, both cell morphology and nuclear morphology changed and presented tumor features such as pleomorphic cells and giant nucleus after cell transformation. However, in this study, no obvious morphology change was found in Balb/c 3T3 cells after 11 weeks PF-EMF exposure.

In addition, to explore the effects of long-term PF-EMF exposure on cell behavior, the cell kinetics including viability, cell cycle and apoptosis were observed. It was found that the cell viability significantly decreased, meanwhile, the percentage of cells in S phase decreased, coupled with an increase in the percentage of cells in the G_2_ phase after 11 weeks PF-EMF exposure. These results suggested that PF-EMF could inhibit cell proliferation in vitro. Our results were consistent with some reports which revealed that 50 Hz ELF inhibited cell proliferation [17,18,19]. However, there are other reports which showed ELF enhanced cell proliferation [12,20]. The contradiction of results above can be ascribed to the difference of experimental conditions, such as exposure parameters [17,21,22] and cell lines [23] As we know, cell cycle related protein CyclinD1 plays a crucial role in the cell cycle transition from G_1_ to S phase. To explore the mechanism that PF-EMF induced cell cycle change in Balb/c 3T3 cells, the protein levels of CyclinD1 was determined in this study. In addition, since PCNA is a key protein in cell proliferation, PCNA was used as another target protein. As showed in Fig. 5, the protein levels of CyclinD1 and PCNA decreased significantly in Balb/c 3T3 cells after 11 weeks exposure compared with sham control, which indicated that these two proteins were involved in PF-EMF induced cell cycle and cell proliferation change.

In case of apoptosis, compared with sham group, the apoptosis level in exposure group showed no significant change which suggested the long-term PF-EMF exposure couldn’t induce apoptosis. These results are consistent with Reipert’ and Ismael’ reports. Reipert found that the apoptosis level in FDCP-mix (A4) cells wasn’t disturbed by 6 microT, 1 mT and 2 mT ELF exposure [24]. Similarly, Ismael reported that 0.4–1 mT, 60 Hz ELF didn’t affect spontaneous apoptosis of mice spleen cells [25].

Since cell transformation is a classic method in determining carcinogens, long-term PF-EMF exposure on cell transformation has been observed in this study. No malignant clones were found in Balb/c 3T3 cells after 11 weeks PF-EMF exposure. In other words, under the condition of this study, long-term PF-EMF exposure could not result in cell malignant transformation. Whether prolong the exposure time or change exposure parameters can initiate cell malignant transformation need further study.

To imitate exposure environment in real life, 2.3 mT was chosen in this study, which was among the occupational exposure intensity range for welders (1~4 mT). Although cell transformation did not occur after 11 weeks PF-EMF exposure, the viability and cell cycle obviously changed. Therefore the possibility of a small and longer-term effect of PF-EMF on human beings thus cannot be ruled out, well designed studies to ascertain the bio-effect of PF-EMF are needed.
